# On the Formation of Nanobubbles in Vycor Porous Glass during the Desorption of Halogenated Hydrocarbons

**DOI:** 10.1038/srep10943

**Published:** 2015-06-05

**Authors:** A. C. Mitropoulos, K. L. Stefanopoulos, E. P. Favvas, E. Vansant, N. P. Hankins

**Affiliations:** 1 Department of Petroleum and Mechanical Engineering, Hephaestus Lab, Eastern Macedonia and Thrace Institute of Technology, Kavala, St. Lucas 65404, Greece; 2 Institute of Nanoscience and Nanotechnology, National Centre for Scientific Research “Demokritos”, Aghia Paraskevi, 153 41, Attica, Greece; 3 Department of Chemistry, Laboratory of Adsorption and Catalysis, University of Antwerp, Universiteitsplein 1, B2610 Wilrijk, Belgium; 4 Department of Engineering Science, The University of Oxford, Parks Road, Oxford OX1 3PJ, UK

## Abstract

Vycor porous glass has long served as a model mesoporous material. During the physical adsorption of halogenated hydrocarbon vapours, such as dibromomethane, the adsorption isotherm exhibits an hysteresis loop; a gradual ascent is observed at higher pressures during adsorption, and a sharp drop is observed at lower pressures during desorption. For fully wetting fluids, an early hypothesis attributed the hysteresis to mechanistic differences between capillary condensation (adsorption) and evaporation (desorption) processes occurring in the wide bodies and narrow necks, respectively, of ‘ink-bottle’ pores. This was later recognized as oversimplified when the role of network percolation was included. For the first time, we present *in-situ* small angle x-ray scattering measurements on the hysteresis effect which indicate nanobubble formation during desorption, and support an extended picture of network percolation. The desorption pattern can indeed result from network percolation; but this can sometimes be initiated by a local cavitation process without pore blocking, which is preceded by the temporary, heterogeneous formation of nanobubbles involving a change in wetting states. The capacity of the system to sustain such metastable states is governed by the steepness of the desorption boundary.

For more than half a century, Vycor porous glass^1^ has been used as a model mesoporous material; according to Brunauer’s classification^2^, it exhibits a type IV adsorption isotherm with an H2 hysteresis loop (see Fig. 1, inset). The early ‘knee’ at low relative pressure is taken to indicate the formation of an adsorbed monolayer of adsorbate molecules. The first models considered that there were two types of pores present, each with a size distribution. The first type were V-shaped, and these filled and emptied reversibly. The second type, known as ‘ink-bottle pores’, had a narrow neck and a relatively wide interior. According to the Kelvin equation^3^, the vapour pressure above the concave meniscus of a wetting liquid decreases with curvature, which is inversely proportional to the meniscus and pore radius. Thus, as relative vapour pressure was increased to one, the gradually increasing steepness of the adsorption branch was taken to reflect the combined effects of monolayer adsorption and gradual capillary condensation in the wide pore interiors with a large distribution of sizes. But, as relative vapour pressure subsequently decreased during the reverse process, the delayed but sharp drop in the desorption branch of the adsorption isotherm was taken to indicate the evaporation from the wide pore bodies via the narrow necks, the latter with a relatively narrow size distribution.

Later on, it was recognised that such a description was oversimplified, and the role of network effects was taken into account. This phenomenological paradigm was in accord with IUPAC recommendations^4^, and the Vycor porous glass has provided a classic example for its experimental demonstration. However, by revisiting our small angle x-ray^5^ and small angle neutron^6^ scattering data (SAXS and SANS), we have concluded that the strong increase in scattered intensity at the commencement of the desorption process may be attributed to the temporary formation of myriad nanobubbles inside the porous glass (for more details of this process, see Fig. 1 and the description in the next section). The extent to which these nanobubbles influence the desorption process and the manner in which they form are both discussed in this report.

It was Ross and co-workers^6^ (of whom KLS is also an author of this report) who first observed this upturn. They interpreted the increase in the scattered intensity as the result of a spaghetti-like percolation cluster induced by mass fractals. However, percolation by its own can not fully explain this upturn, especially at the beginning of the hysteresis area; a driving mechanism is also needed. Moreover, the concept of nanobubbles had not been established or verified at that time. It is only in recent years that an intense research effort^7^,^8^,^9^,^10^,^11^,^12^,^13^ has been devoted to the study of nanobubbles, and especially to their formation and stability, since they appear to last for days or even months. This paradoxical behaviour contradicts the classical view of, for example, the air-water interface, for which the high Laplace pressure inside small bubbles should cause them to dissolve instantly in favour of larger ones (the phenomenon of Ostwald ripening).

While detailed research on pore networks giving rise to type H2 hysteresis loops continues today with novel mesoporous materials^14^,^15^,^16^,^17^,^18^,^19^,^20^,^21^, the effect of various factors on adsorption hysteresis remains an open question. There are two main mechanisms of desorption in such networks: a) when evaporation of the capillary condensate from the pore body occurs after emptying of its neck, the mechanism is known as pore blocking and b) when the pore body empties first, while the pore neck remains filled, the mechanism is known as cavitation. In the first mechanism, the onset of evaporation is associated with a percolation threshold where a continuous path of open pores to the external surface is formed. In the second mechanism, the growth of gas bubbles in the condensed fluid is involved. Naturally, the size of the pore necks is taken to be the factor that determines which mechanism will prevail. When the neck size is small, but not small enough such that the negative capillary pressure will expand the condensed liquid beyond its limiting tensile strength, desorption will obey the pore blocking/percolation mechanism. On the other hand, when the neck size is small enough, the negative capillary pressure exceeds the limiting tensile strength of the liquid and cavitation will succeed.

In addition, other factors such as the surface rugosity of the pore walls may also play a role in the evaporation mechanism, leading to alternative scenarios as extensions of these two cases. For instance, although the formation of nanobubbles of sub-critical size does not lead to a cavitation instability, such nanobubbles may nevertheless assist in the percolation transition.

Rosinberg *et al.*^22^ provided a comprehensive theoretical description of hysteresis during the capillary condensation of gases in mesoporous disordered materials. They suggested that a percolation-dominated draining process does not require the introduction from the outset of a pore-blocking mechanism that limits the accessibility of the filled pores to the outer surface of the material.

Woo *et al.*^23^ have also studied the desorption mechanism of fluids by Monte Carlo simulation on a matrix configuration representative of Vycor porous glass. They concluded that cavitation via nucleation of bubbles inside the pores plays a role in the desorption process. They further suggested the existence of a percolation transition which required neither a pore-blocking mechanism nor cavitation.

By using SANS, Hoinkis and Kuhn^24^ examined *in situ* the sorption of nitrogen at 78 °K on a mesoporous silica glass having a rough internal surface. During desorption, a strong signal at low values of the scattering vector Q was also observed. They interpreted this result in terms of ramified vapour-filled void clusters, and they further speculated that these clusters may originate from a percolation process; this process could occur with or without heterogeneous nucleation or cavitation and the self-similar growth of bubbles.

Bonnet *et al.*^25^ studied the collective effects which occur during adsorption-desorption in Vycor porous glass by light scattering. They concluded that, as temperature increases, a crossover from percolation to cavitation is evident for the evaporation process.

In a review article, Monson^26^ discussed the hysteresis for fluids in mesoporous materials. For disordered pore networks like those in Vycor glass, evaporation from different regions depends upon their spatial location. He suggested that pore blocking and cavitation are key components of the desorption mechanism. Further relevant work includes a noteworthy review by Landers *et al.*^27^ on the characterization of porous materials and one by Thommes and Cychosz on the same topic^28^.

## Results

**Figure 1:**
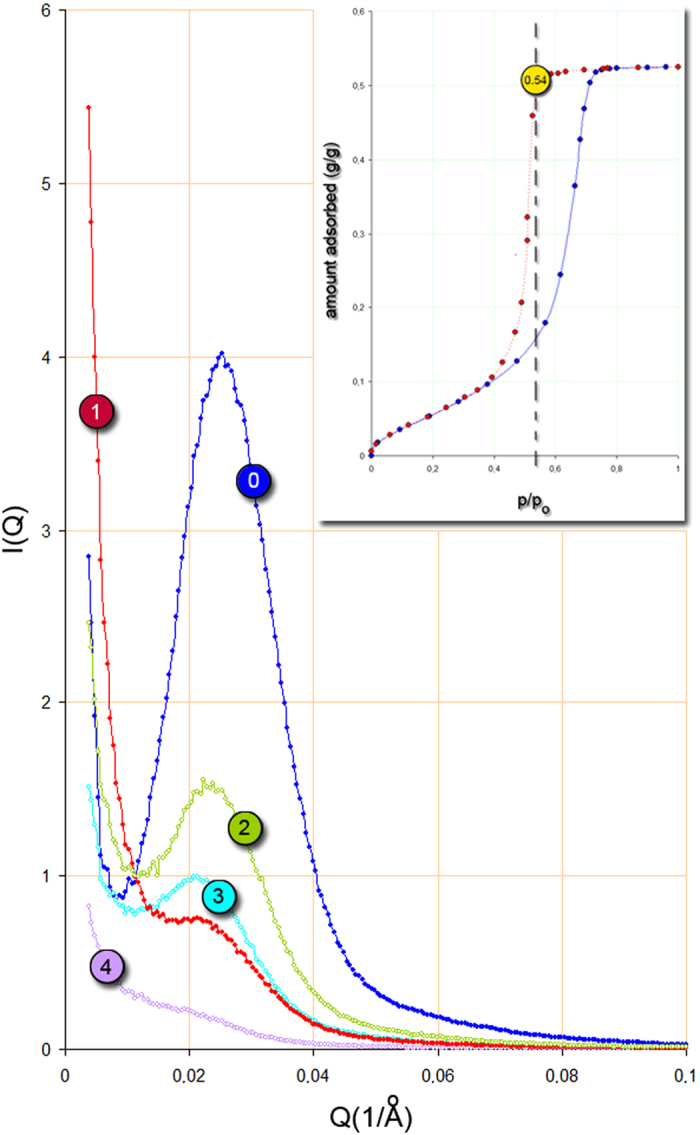
Some small angle X-ray scattering curves of Vycor loaded with CH_2_Br_2_ at various relative pressures on both adsorption and desorption. Curve 0 at p/p_o_ = 0 (dry sample); curve 1 at (p/p_o_)_des_ = 0.54; curve 2 at (p/p_o_)_des_ = 0.49; curve 3 at (p/p_o_)_ads_ = 0.67; and curve 4 at (p/p_o_)_ads_ = 0.77. Notice that in curve 1 the sharp increase of the scattered intensity at low Q is well above the spinodal peak of curve 0; other curves are included for comparison reasons only. The insert shows the adsorption isotherm of CH_2_Br_2_ on Vycor at 293 K. The relative pressure corresponding to curve 1 is indicated at the onset of the desorption process.

Figure 1 shows the SAXS measurements from which the formation of nanobubbles is inferred. The spectrum of dry/empty Vycor (i.e. at p/p_o_ = 0) is characterized by the peak at Q = 0.025 Å^−1^ (curve 0); here, Q = 4πsinθ/λ and 2θ is the scattering angle. On the other hand, during the desorption process, the spectrum of Vycor at (p/p_o_)_des_ = 0.54 (curve 1), which is at the onset of the steep part of the desorption branch (see inset), is characterized at low Q by an increase in the scattered intensity to well above the spinodal peak (compare with curve-0). During adsorption, an adsorptive film is deposited on the pore walls, and eventually all pores are filled with capillary condensate. Since dibromomethane (CH_2_Br_2_) contrast matches the silica matrix, the scattered intensity constantly decreases as p/p_o_ increases. During desorption, however, curve 1 shows that the scattered intensity at low Q, just before the pores empty, increases sharply. This is true, in spite of the fact that, at this relative pressure, the isotherm in the inset indicates that only 3% of the adsorbate has evaporated.

The preceding situation is similar to that of the so-called ‘opacity point’. In much earlier work, a silica gel-water system was found to assume a turbid appearance at a point close to the beginning of the steep part of the desorption isotherm; this was termed by Zsigmondy^29^ as the opacity point. A similar situation was also observed for Vycor porous glass^30^. The glass, which is transparent when saturated, acquires an intense whitish turbidity when a small amount of liquid is removed by evaporation. Haynes and McCaffery^31^ have examined the turbidity in Vycor porous glass with light scattering. They attributed the phenomenon to a non-uniform distribution of full and empty pores large enough to act as Mie scatterers, sustained by hysteresis effects. Based on molecular dynamics calculations of the condensation process within Vycor porous glass, a density redistribution of the adsorbate within the hysteresis region was also concluded by Valiullin *et al.*^32^

As already mentioned, the observed sharp increase in scattering intensity has been confirmed by previous results we have obtained for SAXS and SANS. Furthermore, this outcome is far from universal. Results for other adsorbing and desorbing systems show no such peak; see Figures S1 and S2 in the supplementary information. Such systems are unable to generate the necessary tensile strength in the liquid adsorbate required to create nanobubbles, even by heterogeneous nucleation. As a consequence for these adsorbents, the large sudden jump in scattering intensity upon desorption (as seen in Figure 1) is absent, nor is there evidence of bulk cavitation at the lower knee.

**Figure 2:**
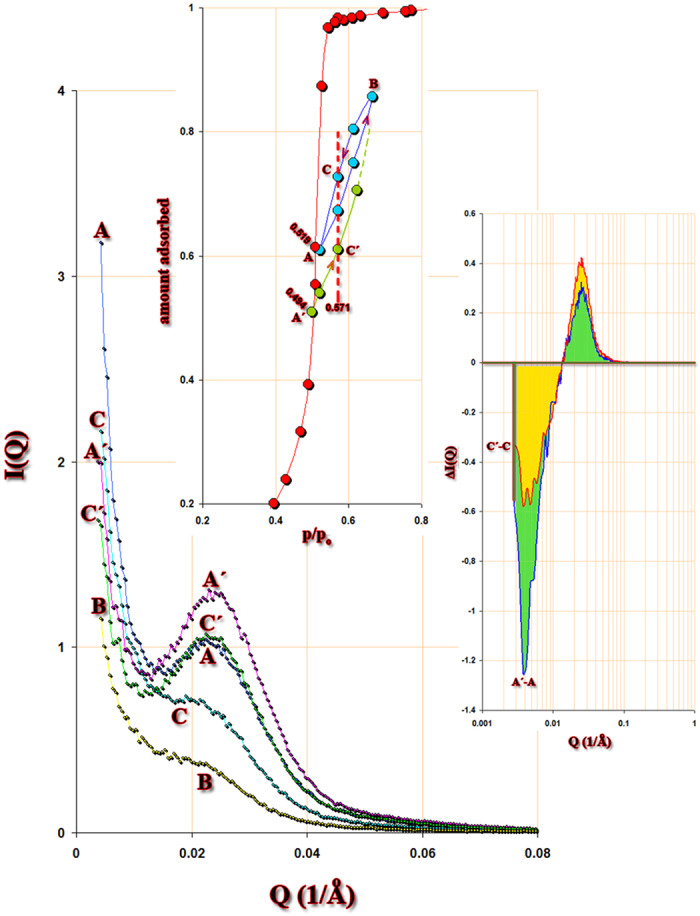
Scanning curves *in situ* with SAXS. Points A and Α΄ are on the desorption boundary. Notice that the low-Q upturn of curve A is about 1.5 times less than the corresponding upturn for curve 1 in Fig. 1. The amount desorbed between points A-A΄and C-C΄ is about the same. A fluctuation in the system may lead to an exchange of capillary condensate in neighbouring regions of the pore network, e.g. C-C΄ where a mass balance is preserved at the same relative pressure. The main figure shows the scattering curves at various values of p/p_o_. Notice that the spinodal peaks at points C΄ and A coincide, although at low Q there is a difference, indicating a redistribution of the capillary condensate which is more clearly presented in the lower insert in terms of ΔI(Q). This redistribution is metastable, but is maintained by hysteresis effects. The magnitude of these effects is dependent on the steepness of the desorption boundary. The sum of the areas (green or yellow) are almost equal to zero, but the system rearranges the amount adsorbed from large clusters to neighboring pores, indicating local cavitation. The upper insert shows the points on the CH_2_Br_2_ desorption isotherm where SAXS measurements are conducted.

To study the metastabilities fixed in Vycor by hysteresis effects, we conducted a scanning of the desorption isotherm to some depth within the hysteresis area, in conjunction with SAXS. Figure 2 shows the results. From point A on the desorption boundary, an adsorption/desorption scanning cycle is performed (ABCA), and from point A΄ an adsorption-only scanning is performed (A΄C΄…). Points A and A΄, which are at different relative pressures, differ between each other by an adsorbed amount which is roughly equal to that between points C and C΄, the latter points being at equal relative pressure. An equivalent situation to this may now be described, as follows. A fluctuation from an initial state A can lead to transient states, e.g. A΄ and B, in adjacent pore regions. After equilibrium is re-established, B moves down to C and A΄ enters the hysteresis loop to C΄, where the two states are in equilibrium at the same p/p_o_. This will result in a redistribution of the capillary condensate within the system, which is clearly illustrated at the lower inset of Fig. 2. In both coloured areas, the sum of negative and positive ΔI(Q) is equal to zero. It is the steepness of the desorption boundary which defines the capacity of the system to maintain such metastable distributions; in a sense, the steeper the boundary curve, the larger the saturation differences that can be sustained.

**Figure 3:**
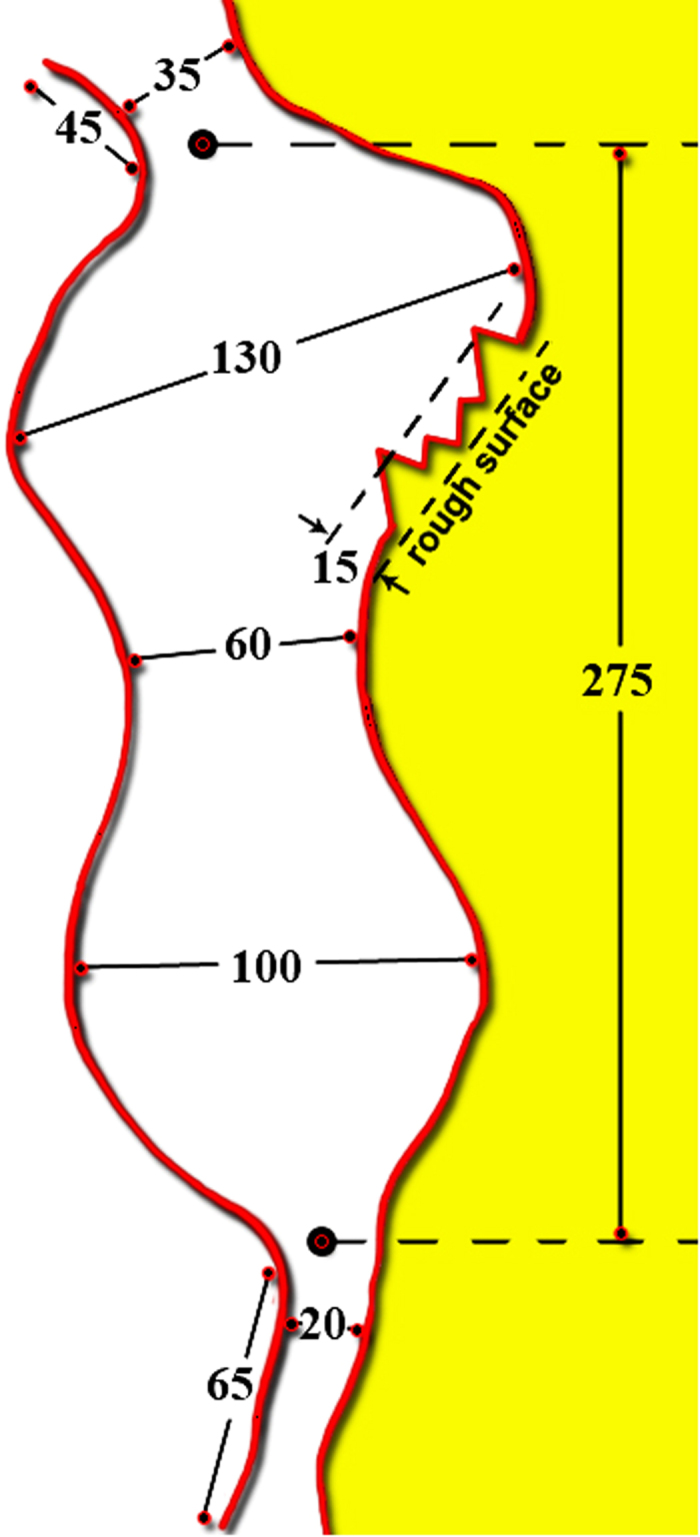
A sketch of a pore which is made from a simulated 2D TEM image of dry Vycor (see Ref. 33); all values are in Ångstroms. For an equivalent single cylindrical pore, the average pore size is about 70 Å and the length 275 Å (for details see the text).

In order to gain a better understanding of our results, we draw a picture of a single pore in Vycor. However, it should be noted that this is only a restrictive case for illustrative purposes; the real pore system in Vycor porous glass is far more complicated, and in some cases the descriptions provided have proven controversial. Based on a simulated 2-D TEM image reported by Kim and Glinka^33^ with the aid of small-angle scattering data, we have drawn Fig. 3 to summarize a number of average-size estimates of various pore features of Vycor porous glass. The pore walls are sinusoidal in profile and define a pore body formed from two cavities with necks at each end. In the middle, where the sinusoidal walls converge, the pore body is narrowed but not as much as in the necks. The length of the pore body is roughly equal to the Bragg spacing, d. This latter is related to the SAXS scattering vector Q corresponding to the characteristic peak of the Vycor porous glass spectrum, via the expression d = 2π/Q; it ranges between 250 and 285 Å. The average pore size is about 70 Å. Furthermore, the pore walls of Vycor porous glass are rough, with a fractal dimension of about 2.3. This roughness has an upper cut-off limit of about 15 Å. CH_2_Br_2_ and Nitrogen BET areas are found to be 80 and 135 m^2^/g, respectively. Other details for the glass may be found elsewhere^34^. Adsorption isotherms for CH_2_Br_2_ and Nitrogen are presented in the supplementary material.

## Discussion

**Figure 4:**
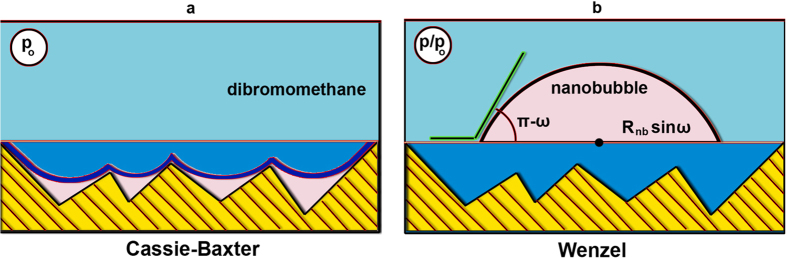
Sketch showing the formation of an interfacial nanobubble: **a**) during adsorption, the surface roughness accommodates an amount adsorbed locally in a Cassie-Baxter wetting state and **b**) during desorption, the tensile force overcomes the free energy barrier in passing from Cassie-Baxter to Wenzel states, resulting in the heterogeneous formation of an INB.

In a previous study^5^, it was found that the roughness of the internal surface of the Vycor porous glass does not rely entirely on its micro-porosity. During the leaching process, a hydrogel layer is deposited on the pore walls. Following drying, this soft hydrogel is converted to an asymmetric xerogel layer which includes cavities, bridges, and bumps conferring a roughness to the surface in a similar manner to that of e.g. a woven textile fibre. Under these circumstances, the adsorbate molecules experience the pore surface as though it consisted of a porous textile of fibres, and so interact with the adsorbent surface according to a Cassie-Baxter^35^ type wetting process (Fig. 4a). However, during desorption, the negative capillary pressure associated with the smaller pores that control the entrance to the pore body result in the exertion of a tensile force on the condensed liquid. Under this force, the adsorbate molecules may find room in the underlying xerogel; nanobubbles are formed and accommodated by the space thus freed. The adsorbate now interacts with the Vycor surface according to a Wenzel^36^ type wetting process (Fig. 4b).

**Figure 5:**
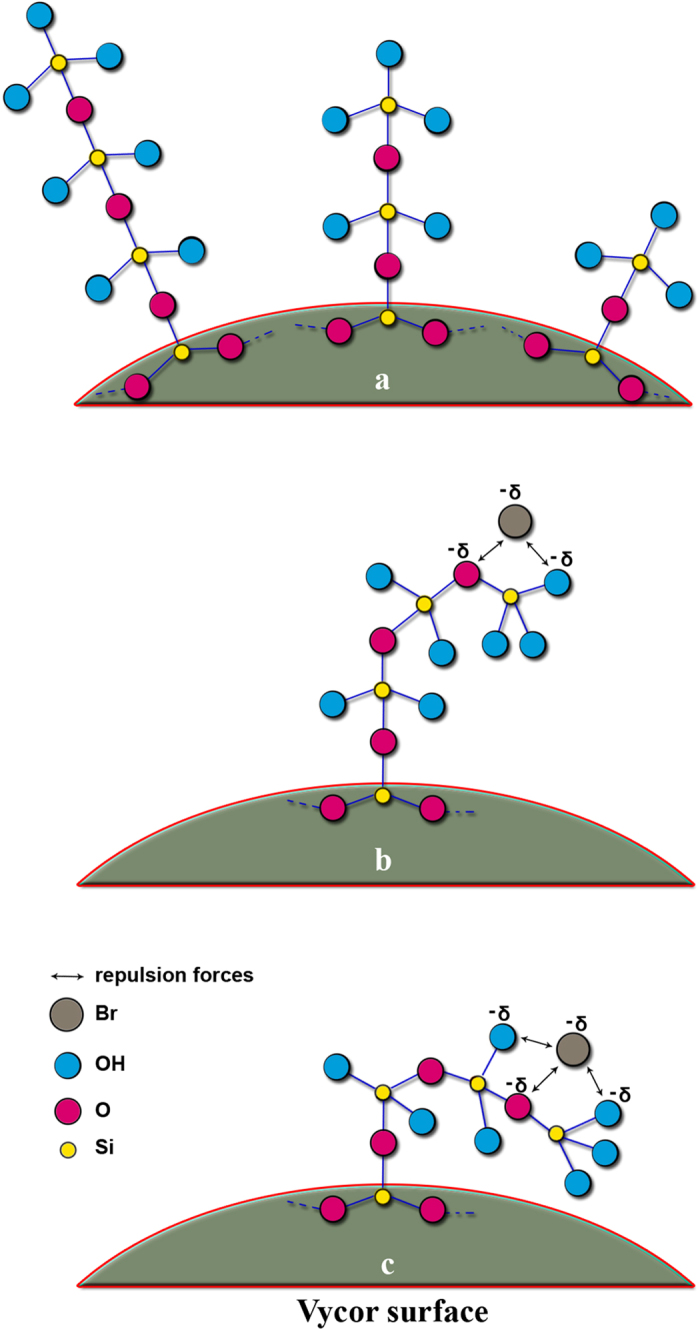
**a**) Examples of possible Si-chains on the Vycor surface (≡Si-O-Si≡ siloxane bonds, ≡Si-OH silanol group); **b** and **c**) possible bending examples of ≡Si-O chains on the Vycor surface due to repulsion forces between Br of CH2Br2 and siloxane/silanol groups.

An ideal chemical model for this wetting transition is presented in Fig. 5. During the synthesis of amorphous Vycor porous glass, important phase separation and phase equilibria effects can take place^37^. A vertical and horizontal Si polymerisation with the chemical post-synthesis treatment will result in a typical pore system for Vycor porous glass, where geometrically different broken siloxane chains and silanol groups are expected at the pore surface, thus explaining its roughness (Fig. 5a). When halogenated hydrocarbons (such as CH_2_Br_2_) are adsorbed at a moderate temperature, e.g. 293 K, on the Vycor surface, the surface siloxane chains will bend towards the surface because of the presence of significant repulsive forces between, on the one hand, the siloxane bonds and the silanol groups (the latter having a basic nature) and, on the other, the electronegative character of the bromine group (Fig. 5b,c). This leads to adsorbed molecules of CH_2_Br_2_ lying on top of bended siloxane chains (the Cassie-Baxter model). During desorption, with a rearrangement of the surface siloxane chains due to rotational, vibrational and migrational effects, nanobubbles can be formed (the Wenzel model).

A similar molecular rearrangement was observed during the adsorption and desorption of alkylamines on clays, which depended on the length of the alkyl chain^38^. On the other hand, such behaviour, which is typical for Vycor porous glass, is very unlikely^39^ to arise in the semi-crystalline mesoporous materials studied by e.g. Voort *et al.*^40^ and Ravikovitch *et al.*^41^, where their model is based entirely on the physical mechanisms of adsorption and desorption for nitrogen, argon and krypton at 77 °K and 87 °K. Furthermore, the adsorption/desorption temperature, together with the surface roughness, the concentration and flexibility of the siloxane chains and the chemical properties of the adsorbate molecules are all very important factors in the behaviour of the desorption process in Vycor porous glass, as described in the proposed alternative model.

We now analyse the energy barrier to nanobubble formation, as follows. The negative pressure or tension (τ) of the capillary condensed liquid at a relative vapour pressure of p/p_o_ is given by^42^,^43^:
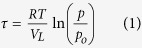
where V_L_ is the molar volume, and T is the isotherm temperature. In the present case, (p/p_o_)_des_ = 0.54 and thus τ = −21.6 MPa. Since p_o_ for CH_2_Br_2_ at 293 K is equal to 4.65 kPa, the correction of Eq.1 for the vapour pressure is negligible. When a nanobubble is formed by homogeneous nucleation, the total energy ΔE^hom^ is the sum of the surface free energy required to form a nanobubble of radius R_nb_ and the work of nanobubble formation (equivalent to the lowering of free energy):
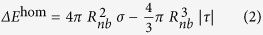
where σ is the surface tension (for CH_2_Br_2_ σ = 40.2 mN/m). This energy reaches a maximum value (ΔΕ^hom^)_max_ = 16πσ^3^/3τ^2^ at the Kelvin radius r_k_ = 2σ/|τ| at a given p/p_o_; above this radius, the bubble formation leads to a lowering of free energy and is thus spontaneous. At p/p_o_ = 0.54, from (1), r_k_ = 37 Å and then (ΔΕ^hom^)_max_ = 2.34 × 10^−18^ J or 579 k_B_T, where k_B_ is the Boltzmann constant and T is the isotherm temperature.

According to nucleation theory the rate of bubble formation is proportional to exp(−ΔΕ_max_/k_B_T). We follow the procedure outlined by Grosman and Ortega^43^ to estimate ΔE_max_ from adsorption isotherm data. Based on the amount adsorbed as measured from the isotherm, and the geometry of a single pore (see Fig 3), we find the energy barrier for nucleation to be ΔE_max_ = 1.56 × 10^−19^ J = 39 k_B_T. According to the expression (ΔΕ^hom^)_max_ = 16πσ^3^/3τ^2^ , this energy value corresponds to a negative pressure of |−84| MPa, which is around four times higher than the actual value of |τ|. Therefore, at p/p_o_ = 0.54, bulk cavitation (via homogeneous nucleation) is unlikely.

However, bubbles can also be produced by heterogeneous nucleation; that is to say, they are formed on the pore walls and particularly rough ones with reduced bubble surface free energy, rather than in the bulk fluid. In this case, the negative pressure of −21.6 MPa which arises at p/p_o_ = 0.54, and where the steep upturn in the scattering intensity at low Q is observed, may qualify for such a local cavitation event. To this end, let us consider the energy required to form a fraction of an interfacial nanobubble (INB) on the Vycor surface, and relate it to the energy involved in passing one mole of condensed liquid from Cassie-Baxter to Wenzel wetting states.

**Figure 6:**
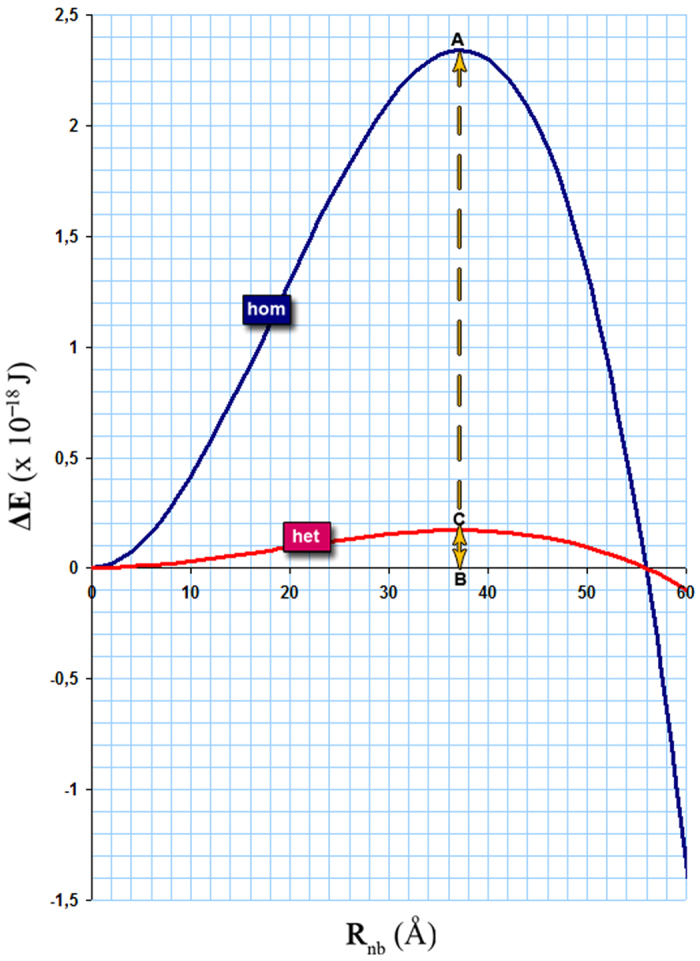
Free energies values for the formation of a nanobubble and an interfacial nanobubble of radius Rnb in CH2Br2 under negative pressure; AB = ΔΕ^hom^ = 2.34 × 10^−18^J and CB = ΔΕ^het^ = 1.56 × 10^−19^ J. Heterogeneous nucleation requires about the 1/15^th^ of homogeneous nucleation energy to form an INB of R_nb_ = 37 Å with lateral size α/2 = R_nb_ sin(π−133^o^) = 27 Å.

The maximum value of (ΔE^het^)_max_ corresponding to the heterogeneous nucleation of an INB of critical size R_c_, is given by^44^ (ΔE^het^)_max_ = Φ(m) × (ΔE^hom^)_max_ where Φ(m) = ¼(2 + m)(1 − m)^2^, m = cos(π − ω) and ω is the contact angle (taken on the same side as the liquid). For (ΔΕ^het^)_max_ = ΔΕ_max_ and ω = 133^ο^, an INB of lateral size α/2 = 27 Å and R_c_ = r_k_ = 37 Å is concluded. Fig. 6 shows the results for both types of nucleation.

The free energy barrier (ΔG_cw_) in moving from a Cassie-Baxter to a Wenzel wetting state is highly dependent on the height of the surface pillars and the liquid contact angle^45^. For the adsorption of CH_2_Br_2_ on Vycor porous glass, an autophobic behaviour requiring the use of a finite angle of contact was previously suggested^46^. For pillar heights less than a critical value (about 13.5 Å), the Wenzel wetting state prevails. For pillars higher than this critical height, the Cassie-Baxter state is metastable. Coexistence of Wenzel and Cassie-Baxter states is thus possible, depending on the local characteristics of the pore wall roughness. In the case of Vycor porous glass, where the characteristic height of roughness features is about 15 Å, is ΔG_cw_ not more than 1 k_B_T. It is noted that the strength of a hydrogen bond with halogenated groups is about 160 J/mol. In any event, ΔG_cw_ is much less than the energy of heterogeneous formation of an INB.

The effect of pore geometry on the conditions for cavitation has also been discussed by Ravikovitch and Neimark^47^. They have concluded that the cavitation pressure in spherical pores is higher than that for cylindrical pores. That is, the lower closure point of the hysteresis loop depends not only on the adsorbate and the temperature but also on the pore geometry. Although this is an important conclusion, it is interesting to note that this is not a property of the solid. It is the liquid which has the intrinsic property of taking on the shape of the vessel that contains it; there is no physicochemical interaction, in this reasoning, between bulk liquid and solid. To show this subtle difference, let us provide the following example. Everett^48^ introduced a descriptor for the geometry of the pores in a solid using a numerical factor γ, such that:
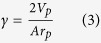
where V_p_ is the pore volume, A is the solid surface area and r_p_ is the mean pore size. This would mean that for non-intersecting cylindrical capillaries of uniform size which are open at both ends γ = 1, whereas for closed spherical pores γ = 2/3; i.e. for equal pore volumes, A_cyl_ < A_sph_. However, for the liquid column which is accommodated within this cylindrical pore (assuming flat menisci), the surface area will always be greater than that for the spherical blob, as is expected; i.e. for equal volumes the former will always be less ‘bulky’ than the latter. Since homogeneous nucleation takes place within the volume of the bulk liquid, it may be concluded that (p^cav^/p_o_)_cyl_ < (p^cav^/p_o_)_sph_.

During heterogeneous nucleation, the opposite situation arises. In this case, there is an interaction between the solid/liquid interface, which is readily inferred from the energy required to form an INB:
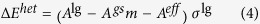
where A^lg^ and A^gs^ are respectively the areas of the liquid/gas and gas/solid interfaces, A^eff^ is the effective area defined by dA^eff^ = ∫C^lg^δV^lg^ and C^lg^ is the curvature of the liquid/gas interface. Note that the term in parenthesis is the Gauss equation^49^, and by assuming that the contact angle is independent of the volume, Eq.4 can be transformed to:
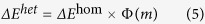


Now, the CH_2_Br_2_/Vycor system has a surface-to-volume ratio of about 4 × 10^8^ m^−1^ whereas the N_2_/Vycor system has a value of 5 × 10^8^ m^−1^. The liquid-like adsorbed film on the pore walls, which protects the interior of the capillaries from surface contaminants and irregularities that otherwise may serve as nucleation sites^19^, is rather shallow and at a much higher temperature in the case of CH_2_Br_2_ compared to that of N_2_. The surface roughness will increase this autophobicity, and thus will increase the probability for a local cavitation event at higher p/p_o_.

However, in heterogeneous nucleation a local cavitation event by its own is not critical if it cannot propagate within the pore network. When pores are unconnected or loosely connected, heterogeneous nucleation in a small fraction of the pores may not have an effect on the macroscopic properties of the medium; the event will be confined by the pore boundaries. However, in Vycor, there are about 3 × 10^17^ pores/g which are fully interconnected. Therefore, a local cavitation event in one of the pores may spread to some extent to neighboring pores, thus making a noticeable difference in e.g. the scattering properties of the medium.

**Figure 7:**
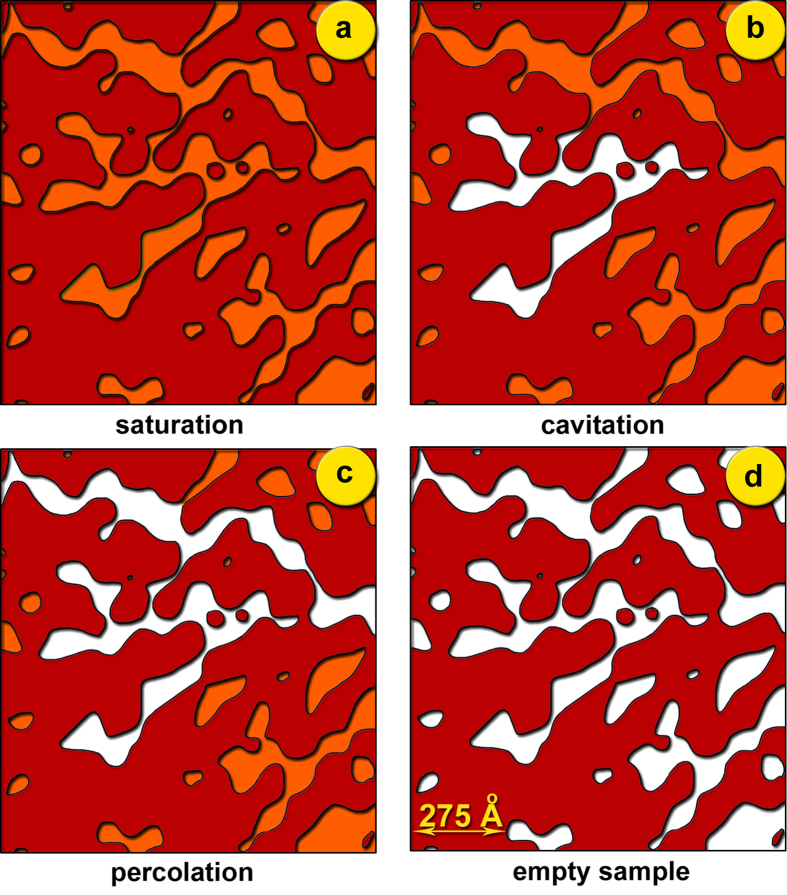
Illustration of the desorption mechanism in Vycor porous glass: **a**) the system is at the saturation point; **b**) local cavitation events result in large clusters of open neighboring pores that act as e.g. Mie scatterers, hence the upturn in the scattered intensity at low Q (see curve 1 of Fig. 1); **c**) the cavitation events propagate a percolation transition; and **d**) all pores are empty.

At very low values of Q, the scattering is generally determined by large entities (ones with a length scale larger than 1,000 Å). We explain the large rise in scattered intensity at low Q as follows. At early stages of the desorption process, nanobubbles with sizes of the order of about 50–60 Å result in an heterogeneous cavitation event which occurs locally, rather than globally, within the porous network. This localized cavitation event spreads towards adjacent network portions and, from there, develops into a vapour cluster by coalescence; this is large enough to give the strong upturn in the scattering spectrum. Percolation without the need for a pore blocking mechanism may thus develop. However, although initially the pore blocking mechanism is not actively involved in this process, it still plays an important role in the desorption process. This is to govern the spatial extent of cavitation events by defining the steepness of the desorption boundary and consequently the capacity of the system to lock them into metastable equilibria. Furthermore, hysteretic behaviour may arise as a consequence of surface interactions and can be explained without additional assumptions about the pore structure or on the detailed shapes of the liquid menisci^50^. Figure 7 illustrates a schematic for the progressive desorption process within the porous system. Further work with adsorbents of similar surface nature but different pore size is underway.

## Methods

In this study, we present *in-situ* measurements on the adsorption of dibromomethane (CH_2_Br_2_) onto Vycor porous glass using small angle x-ray scattering. Dibromomethane is able to ‘contrast match’ with amorphous silica; in this way, when a set of glass pores is filled with condensed CH_2_Br_2_ liquid, they will cease to act as an X-ray scatterer and only the remaining empty pores will produce a measurable scattering intensity. It should be noted, however, that the sample cell which facilitates this adsorption process in conjunction with SAXS measurements may introduce an error in the temperature (held at 293 K), and consequently in the relative pressure, of the order of ±0.2 K and ±0.01, respectively.

Small angle x-ray scattering measurements were performed on a JJ X-ray system (Denmark) equipped with a Rigaku Helium-3 detector and a Cu (λ = 1.54098 Å) rotating anode operated at 40 kV and 40 mA. The sample-to-detector distance and the beam centre were precisely determined by calibration with the Ag–behenate standard (d001 = 58.38 Å). Scattering data were corrected for dark current and empty tube scattering. The Q-range is varied approximately from 0.004 to 0.11 Å^−1^. Nitrogen adsorption measurements at 77 K were performed using an Autosorb-1 static volumetric system (Quantachrome Instruments). Dibromomethane adsorption-desorption isotherms were conducted gravimetrically at 293 K by means of an Intelligent Gravimetric Analyser (IGA, Hiden Isochema). In both adsorption experiments the samples were outgassed overnight at 473 K under high vacuum.

Although further details on the experimental procedure have been published elsewhere^5^,^6^, this is a novel type of experiment and a first time to our knowledge of scanning the hysteresis loop in conjunction with SAXS. Since the properties of the glass may vary between samples from different lots^34^, it is worth noting that our new and our old data, obtained at different time periods and places and with different Vycor samples, chemicals, and instruments, all reflect the same result; that is, an intensity increase at very low Q, well above the spinodal peak.

## Additional Information

**How to cite this article**: Mitropoulos, A. C. *et al.* On the Formation of Nanobubbles in Vycor Porous Glass During the Desorption of Halogenated Hydrocarbons. *Sci. Rep.* **5**, 10943; doi: 10.1038/srep10943 (2015).

## Supplementary Material

Supplementary Information
