# Supplementary material for: On the Formation of Nanobubbles in Vycor Porous Glass during the Desorption of Halogenated Hydrocarbons
Source: Sci Rep. 2015 Jun 5;5:10943. doi: 10.1038/srep10943 (PMC4650640; doi:10.1038/srep10943)
Supplement: Supplementary Information [file srep10943-s1.pdf]

ON THE FORMATION OF NANOBUBBLES IN VYCOR POROUS GLASS DURING THE DESORPTION OF HALOGENATED HYDROCARBONS

A. C. Mitropoulos[1], K. L. Stefanopoulos[2], E. P. Favvas[2], E. Vansant[1,3] & N. P. Hankins[4*]

[1]Department of Petroleum and Mechanical Engineering, Hephaestus Lab, Eastern Macedonia and Thrace Institute of Technology, Kavala, St. Lucas 65404, Greece. [2]Institute of Nanoscience and Nanotechnology, National Centre for Scientific Research "Demokritos", Aghia Paraskevi, 153 41, Attica, Greece. [3]Department of Chemistry, Laboratory of Adsorption and Catalysis, University of Antwerp, Universiteitsplein 1, B2610 Wilrijk, Belgium. [4]Department of Engineering Science, The University of Oxford, Parks Road, Oxford OX1 3PJ, UK.

*Corresponding author. E-mail address: nick.hankins@eng.ox.ac.uk

SUPPLEMENTARY INFORMATION

Figure S1 and S2 present scattering results for the adsorbate dibromomethane during desorption on the mesoporous silica adsorbents SBA-15 and MCM-41. These materials have a highly ordered porous structure and, in the case of MCM-14, the pores are unconnected. The adsorption and desorption isotherms exhibit a H1 rather than a H2 hysteresis loop. Such materials are not fractal, and the pores do not have necks; as a result, they are unable to generate the necessary tensile strength in the liquid adsorbate required to create Nanobubbles, even with heterogeneous nucleation. As a consequence for these adsorbents, the large sudden jump in scattering intensity upon desorption (as seen in Figure 1) is absent.

Figure S3 presents the adsorption isotherm of CH_2Br_2 on Vycor 7930 at 293 K, and Figure S4 presents the adsorption isotherm of Nitrogen on Vycor 7930 at 77 K.

FIGURE LEGEND

Figure S1. Desorption of CH_2Br_2 from SBA-15 in situ with SAXS. No upturn is shown.

Figure S2. Desorption of CH_2Br_2 from MCM-41 in situ with SAXS. No upturn is shown.

Figure S3. CH_2Br_2 adsorption isotherm on Vycor 7930 at 20 ^{o}C. Amount adsorbed in g/g.

Figure S4. N_2 adsorption isotherm on Vycor at 77 K. Amount adsorbed in cm^3/g (STP).

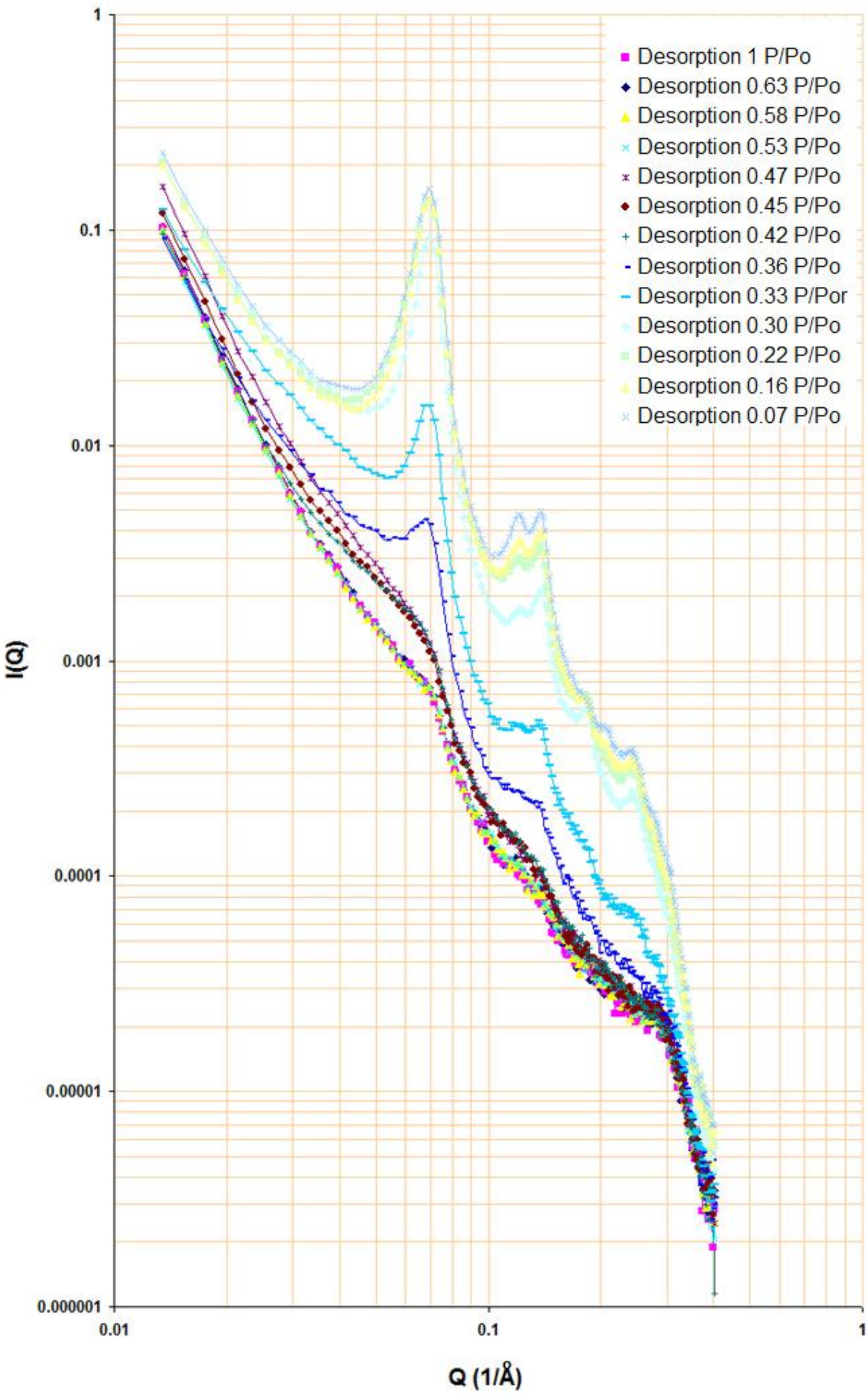

Fig.S1. Desorption of CH_2Br_2 from SBA-15 in situ with SAXS. No upturn is shown.

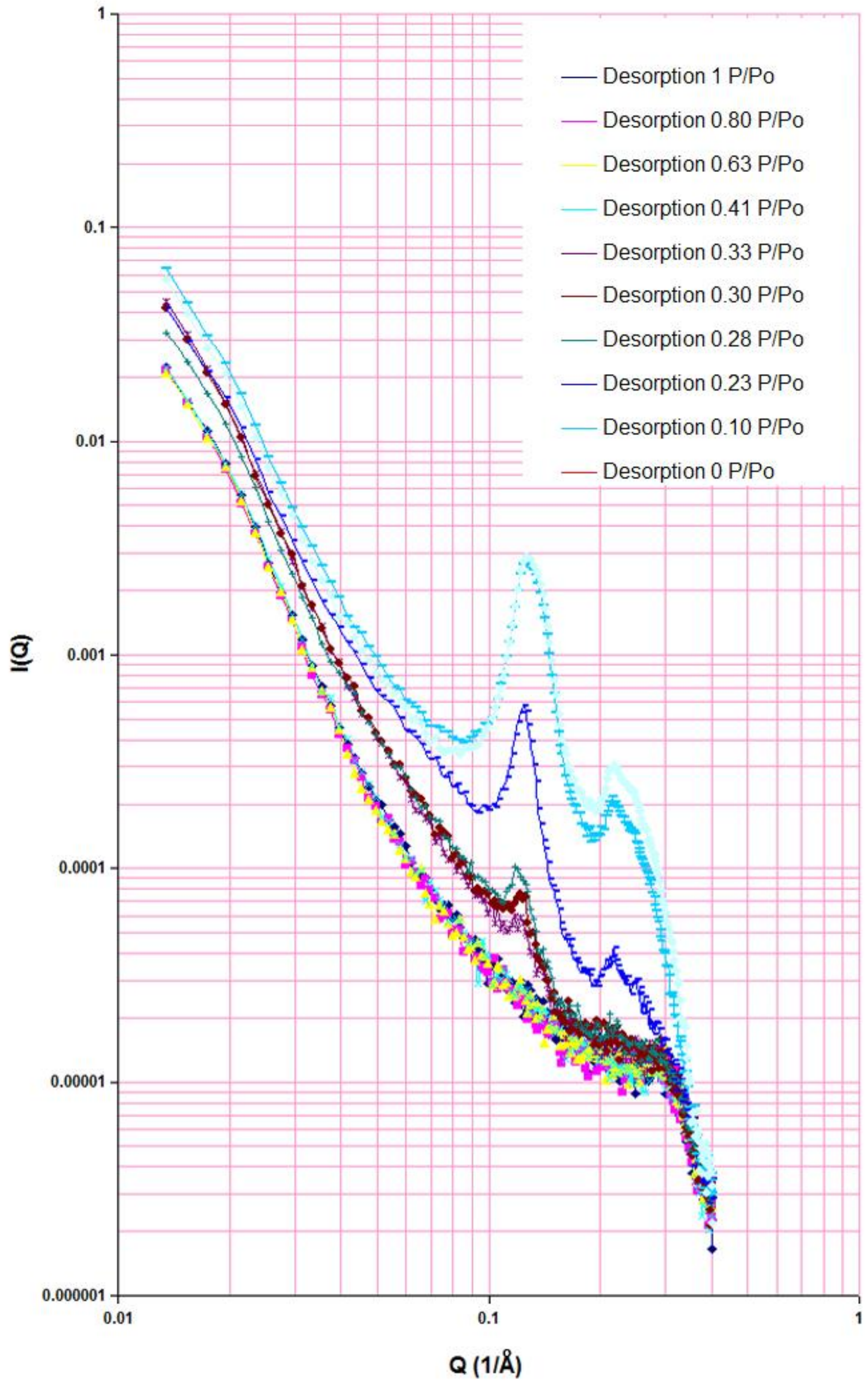

Fig.S2. Desorption of CH_2Br_2 from MCM-41 in situ with SAXS. No upturn is shown.

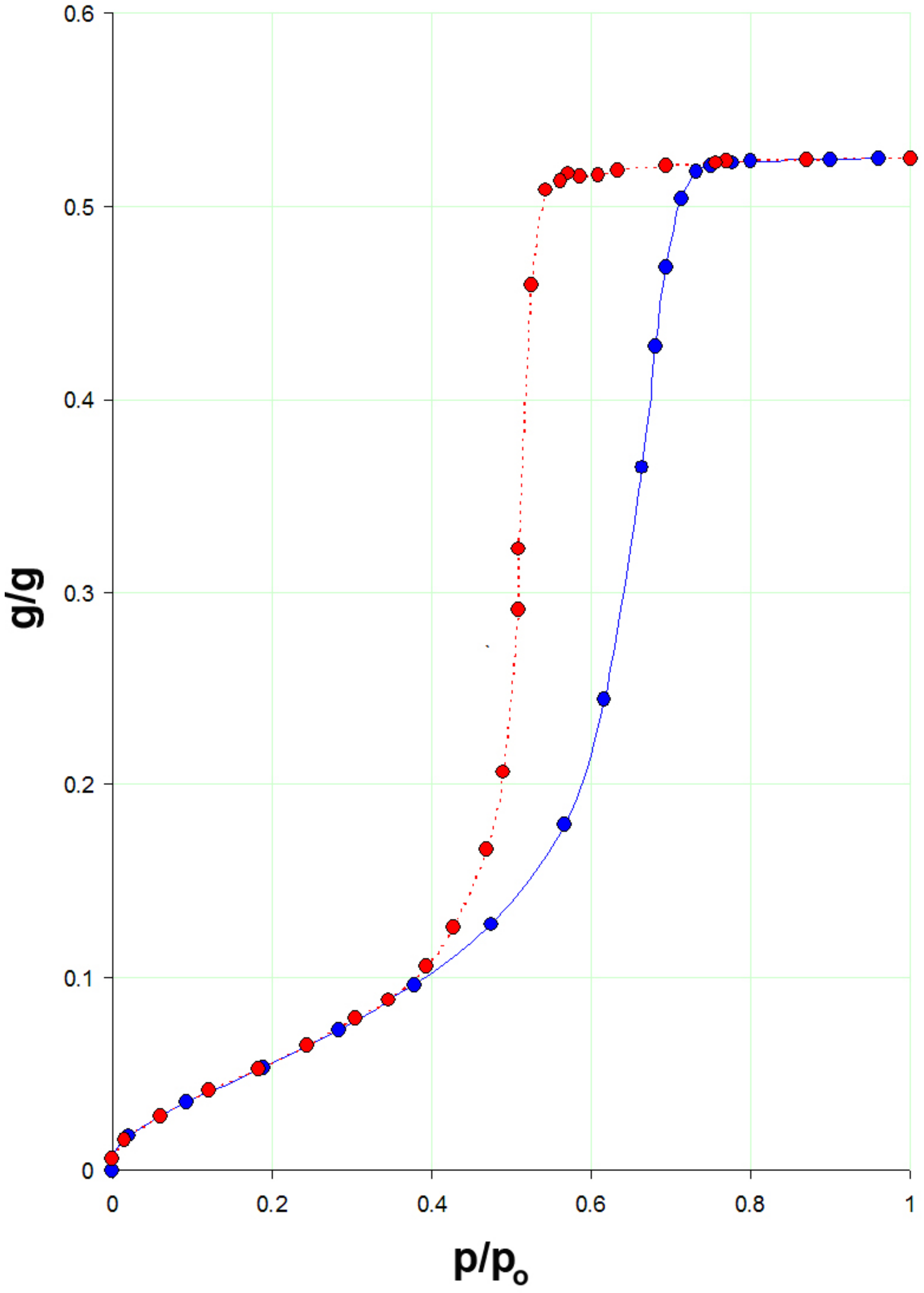

Fig.S3. CH_2Br_2 adsorption isotherm on Vycor 7930 at 20 ^oC. Amount adsorbed in g/g.

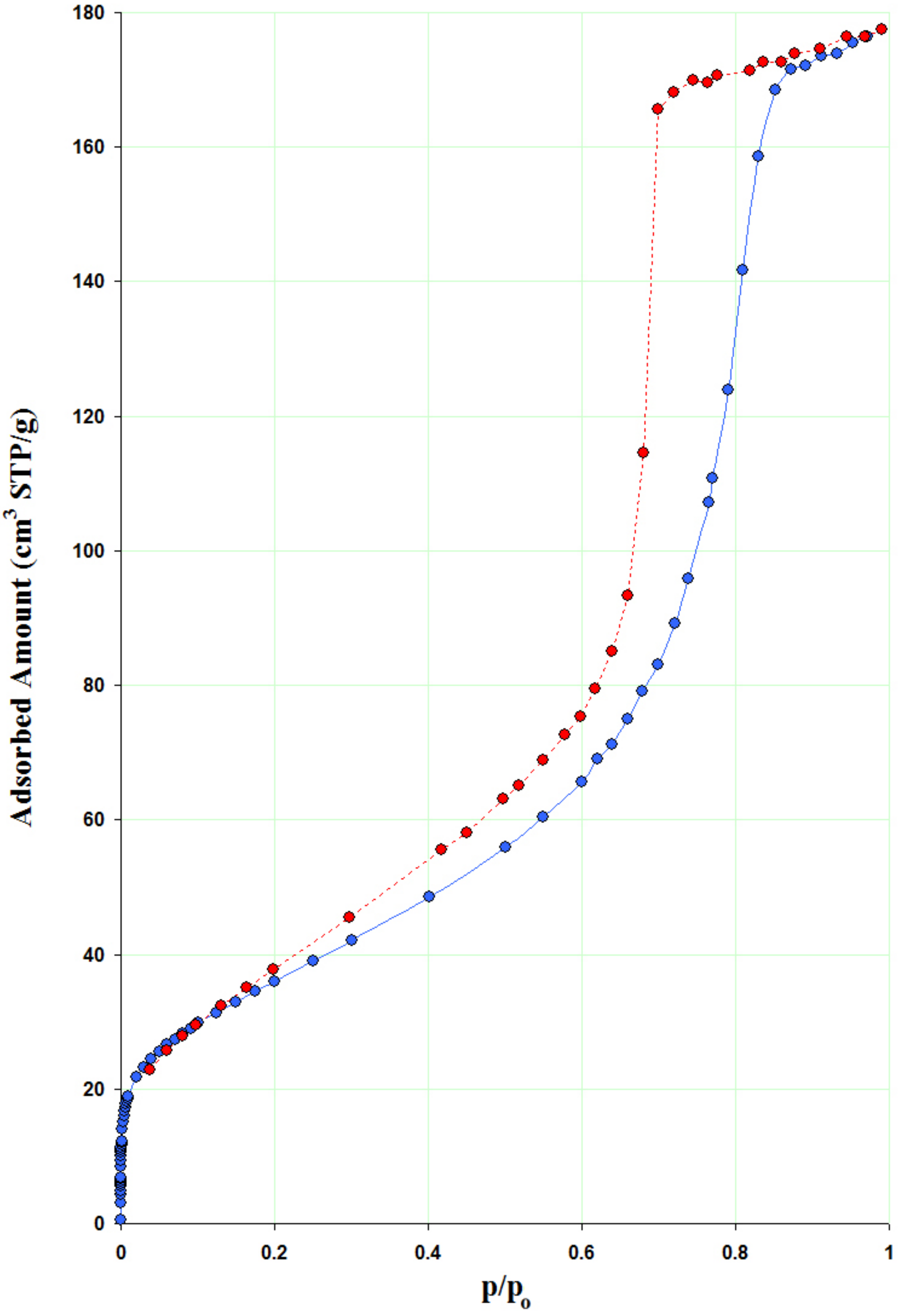

Fig.S4. N_2 adsorption isotherm on Vycor at 77 K. Amount adsorbed in cm^3/g (STP).
